# Regulation of fatty acid desaturase- and immunity gene-expression by *mbk-1/DYRK1A* in *Caenorhabditis elegans*

**DOI:** 10.1186/s12864-021-08176-y

**Published:** 2022-01-04

**Authors:** Hildegard I. D. Mack, Jennifer Kremer, Eva Albertini, Elisabeth K. M. Mack, Pidder Jansen-Dürr

**Affiliations:** 1 Institute for Biomedical Aging Research, University of Innsbruck, Rennweg 10, 6020 Innsbruck, Austria; 2 Department of Hematology, Oncology and Immunology, Philipps-University Marburg, and University Hospital Giessen and Marburg, Baldingerstrasse, 35032 Marburg, Germany

**Keywords:** Lifespan regulation, Aging, Insulin-like signaling, Germline stem cells, RNA-seq, Fatty acid desaturation, Pathogen defense, Hydrogen sulfide

## Abstract

**Background:**

In the nematode *Caenorhabditis elegans*, longevity in response to germline ablation, but not in response to reduced insulin/IGF1-like signaling, is strongly dependent on the conserved protein kinase minibrain-related kinase 1 (MBK-1). In humans, the MBK-1 ortholog DYRK1A is associated with a variety of disorders, most prominently with neurological defects observed in Down syndrome. To better understand *mbk-1*’s physiological roles and their dependence on genetic background, we analyzed the influence of *mbk-1* loss on the transcriptomes of wildtype and long-lived, germline-deficient or insulin-receptor defective, *C. elegans* strains by RNA-sequencing.

**Results:**

*mbk-1* loss elicited global changes in transcription that were less pronounced in insulin-receptor mutant than in germline-deficient or wildtype *C. elegans*. Irrespective of genetic background, *mbk-1* regulated genes were enriched for functions in biological processes related to organic acid metabolism and pathogen defense. qPCR-studies confirmed *mbk-1* dependent induction of all three *C. elegans* Δ9-fatty acid desaturases, *fat-5*, *fat-6* and *fat-7*, in wildtype, germline-deficient and insulin-receptor mutant strains. Conversely, *mbk-1* dependent expression patterns of selected pathogen resistance genes, including *asp-12*, *dod-24* and *drd-50*, differed across the genetic backgrounds examined. Finally, *cth-1* and *cysl-2*, two genes which connect pathogen resistance to the metabolism of the gaseous messenger and lifespan regulator hydrogen sulfide (H_2_S), were commonly suppressed by *mbk-1* loss only in wildtype and germline-deficient, but not in insulin-receptor mutant *C. elegans*.

**Conclusion:**

Our work reveals previously unknown roles of *C. elegans mbk-1* in the regulation of fatty acid desaturase- and H_2_S metabolic-genes. These roles are only partially dependent on genetic background. Considering the particular importance of fatty acid desaturation and H_2_S for longevity of germline-deficient *C. elegans*, we propose that these processes at least in part account for the previous observation that *mbk-1* preferentially regulates lifespan in these worms.

**Supplementary Information:**

The online version contains supplementary material available at 10.1186/s12864-021-08176-y.

## Introduction

*Caenorhabditis elegans mbk-1* (minibrain-related kinase 1) encodes an evolutionarily conserved dual-specificity tyrosine-regulated kinase (DYRK) orthologous to *Drosophila melanogaster minibrain* and human *DYRK1A/B* [1–4]. Human *DYRK1A* maps to chromosome 21q22.13 in the Down syndrome critical region, and its overexpression has been implicated in the neurological defects associated with this disorder [5, 6]. Inhibitory single nucleotide variants, small insertions/deletions or complete deletion of one *DYRK1A*-allele in patients or in mice also cause intellectual disability and other abnormalities, highlighting the sensitivity of DYRK1A-activity to gene dosage [7, 8]. *DYRK1A* is expressed in neuronal and non-neuronal tissues during development and adulthood [2, 9] and has been described to contribute to tumor development, both as an oncogene and as a tumor suppressor [2]. Indeed, individuals with Down syndrome display an elevated risk for certain types of childhood leukemia, but a lower risk for solid tumors across all ages [10, 11].

*C. elegans mbk-1* differs from its related kinase *mbk-2* (*DYRK2*) and from a more distant family member, *hpk-1* (*HIPK2*) in its expression and subcellular localization patterns, as well as in its physiological role [3]. MBK-1 is expressed in all somatic cells during development and adulthood and localizes predominantly to the nucleus [3]. Overexpression of *mbk-1* impairs olfactory behavior, paralleling the *DYRK1A*-overexpression induced neurological defects observed in other species [3]. On the other hand, *mbk-1* inactivation does not cause obvious phenotypic alterations under standard culture conditions [3]. Yet, upon exposure to *Pseudomonas aeruginosa* strain PAO1, *mbk-1* is required for protecting *C. elegans* from fast killing by this pathogen [12]. Moreover, *mbk-1* loss strongly reduces longevity in response to germline ablation, while exerting smaller effects on longevity in response to reduced insulin-like signaling, or on wildtype lifespan [13]. The mechanisms by which *mbk-1* promotes pathogen defense and longevity have not been fully elucidated.

Reduced activity of the conserved insulin/IGF1-(like) signaling pathway promotes longevity from *C. elegans* to mammals [14]. Similarly, loss of germline stem cells (GSCs) extends lifespan in *C. elegans* and in *Drosophila melanogaster*, and castration has been associated with longevity in men, suggesting that germline signals also constitute a wide-spread principle of lifespan regulation [15–18]. In *C. elegans*, longevity in response to *daf-2* (insulin/IGF1-like receptor) inhibition and GSC ablation differ in their requirement for certain regulators, such as *mbk-1*, *kri-1* and *tcer-1* [13, 19, 20]. On the other hand, both pathways depend on a common set of conserved key transcription factors, including DAF-16 (FOXO), HLH-30 (TFEB), HSF-1 (HSF1) and SKN-1 (NRF2) [21]. In germline (GSC)-deficient, but not in *daf-2* mutant *C. elegans*, *mbk-1* contributes to full induction of *daf-16* target genes, consistent with the model that *mbk-1* exerts at least a part of its lifespan-modulatory activity through DAF-16 [13]. Of note, a DYRK1A phosphorylation site on FOXO1 appears to be conserved between *C. elegans* and humans [13, 22].

In this study, we aimed to better understand the mechanisms through which *mbk-1* preferentially modulates *C. elegans* lifespan in response to GSC deficiency. Towards this goal, we examined the effect of *mbk-1* loss on stress resistance and gene transcription of normal and long-lived, wildtype, germline-deficient or *daf-2(−)* mutant *C. elegans* strains, in functional assays and by RNA-sequencing.

## Results

### Loss of *mbk-1* decreases heat and oxidative stress resistance in germline-deficient worms

**Fig. 1:**
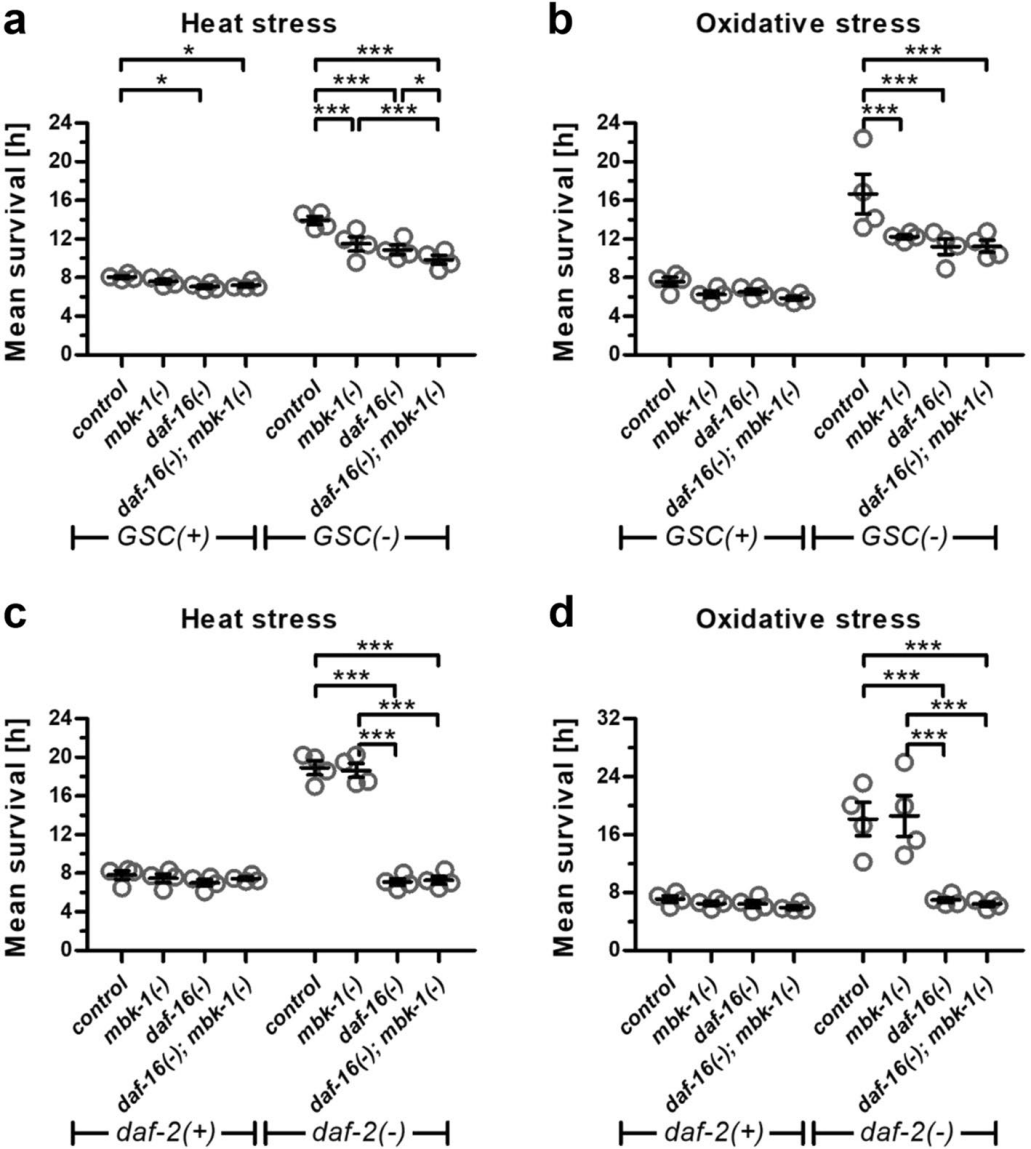
Loss of *mbk-1* decreases stress resistance of germline-deficient *C. elegans.* Strains of the genetic backgrounds indicated, carrying loss of function mutations in *daf-16* and/or *mbk-1* or no additional mutation (control) were subjected to heat **(a, c)** or oxidative stress **(b, d)** and survival was monitored. Data represent mean survival ± SEM from 4 biological replicates of the experiment. Statistical significance of survival differences between groups was determined by repeated measures two-way ANOVA with Bonferroni post tests (* *p* < 0.05, ** *p* < 0.01, *** *p* < 0.001). See Additional file 1: Table S1 for complete statistical analysis

We previously reported that the longevity of GSC deficient *C. elegans* is strongly dependent on *mbk-1* [13]. Conversely, long-lived *daf-2(−)* or wildtype worms do not, or only to a minor extent, require *mbk-1* for lifespan regulation [13]. As changes in longevity are frequently accompanied by changes in resistance to environmental stress such as heat or oxidative stress [23], we examined how *mbk-1* influences these properties in *GSC(−), daf-2(−)* and corresponding *GSC(+)* or *daf-2(+)* control worms (cf. Methods). Upon exposure to heat stress, the loss of function allele *mbk-1(pk1398)* (hereafter referred to as *mbk-1(−)*; [3]), consistently reduced survival of *GSC(−)* worms, while a more moderate, or no reduction of survival was observed in *daf-2(−)* and in *GSC(+)/daf-2(+)* worms (Fig. 1a, c, Additional file 1: Table S1). Oxidative stress resistance on the other hand was consistently decreased in *mbk-1(−)* worms in *GSC(−), GSC(+)* and *daf-2(+)*, but not in *daf-2(−)* background (Fig. 1b, d, Additional file 1: Table S1). Loss of *daf-16* produced a statistically significant decrease in survival in response to each of the two stressors in all genetic backgrounds in all repetitions of the experiment, although effects were generally smaller in *GSC(+)/daf-2(+)* than in long-lived *GSC(−)* or *daf-2(−)* worms [24] (Additional file 1: Table S1). Yet, when *daf-16* and *mbk-1* loss were combined, further reduction of survival was consistently observed only in heat-stressed *GSC(−)* worms (Fig. 1; Additional file 1: Table S1). Collectively, these results are consistent with and expand the concept [13] that *mbk-1* activity is particularly important for *GSC(−)* physiology and longevity. Moreover, our data rise the possibility that *mbk-1* exerts its lifespan- and stress modulatory function in these worms through both, *daf-16* dependent and –independent mechanisms.

### Identification of differentially expressed genes upon loss of *mbk-1*

**Fig. 2:**
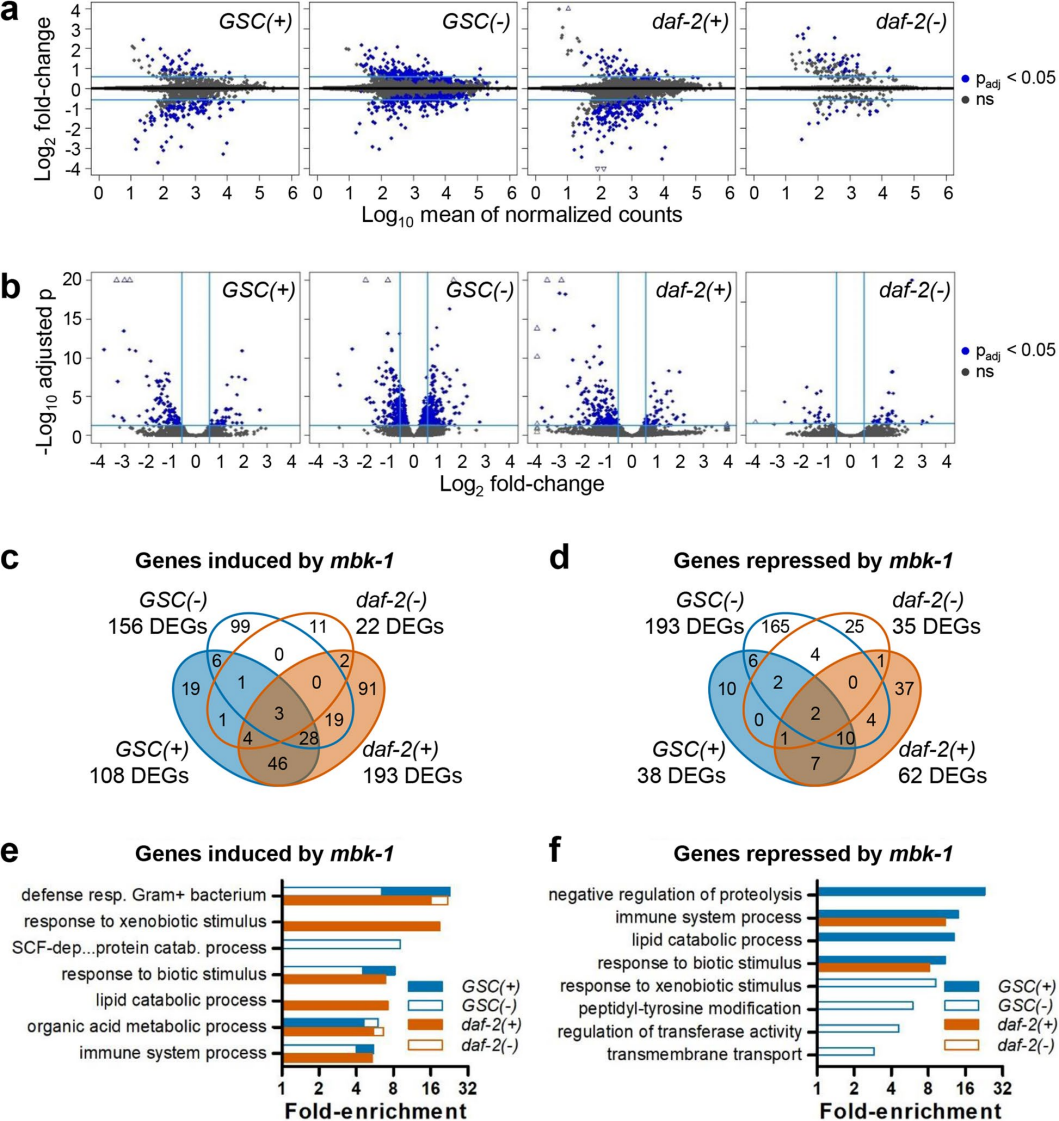
Global changes in gene expression upon *mbk-1* loss in wildtype and long-lived *C. elegans.* **a** and **b** Differential gene expression between *mbk-1(−)* and *mbk-1(+)* worms in the backgrounds indicated, visualized in MA-plots **(a)** and Volcano plots **(b)**. Light blue lines indicate |Log_2_FC| > 0.58 and p_adj_ < 0.05, open triangles indicate data points beyond axis limits. **c** and **d** Overlap of *mbk-1* induced **(c)** and *mbk-1* repressed genes **(d)** from the backgrounds indicated visualized by Venn-diagrams. See Table S4 for statistical analysis. **e** and **f** Biological processes enriched (q < 0.05) among *mbk-1* induced **(e)** and *mbk–1* repressed genes **(f)** in the backgrounds indicated. See Table S6 for complete analysis

To identify genes whose expression is regulated in an *mbk-1* dependent manner during adulthood, we performed whole transcriptome RNA-sequencing (RNA-seq) on *mbk-1(+)* and *mbk-1(−)* worms. To further account for the influence of genetic background on *mbk-1* function, these experiments were conducted in *GSC(−)*, *daf-2(−)* and in *GSC(+)* and *daf-2(+)* control strains. Downstream analyses focused on differentially expressed genes (DEGs) that displayed an expression fold-change (FC) difference > 1.5 between the strains compared. Only when comparing *GSC(−)* to *GSC(+)* worms, this FC-threshold was set to 4 (cf. Ref. [25]). Comparison of DEGs in *daf-2(−)* vs. *daf-2(+)*, and in *GSC(−)* vs. *GSC(+)* worms revealed highly significant overlaps with published RNA-seq studies [25–27], thus validating our data (Additional file 1: Tables S2, 3; Additional file 2: Table S9; Additional file 3: Fig. S1). Globally, changes in gene expression upon *mbk-1* loss were more limited in *daf-2(−)* than in *GSC(−)* or *GSC(+)/daf-2(+)* control worms. (Fig. 2a, b). Genes downregulated upon *mbk-1* loss (*mbk-1* induced genes) from *GSC(+)*, *GSC(−)*, *daf-2(+)* and *daf-2(−)* strains significantly overlapped with each other, and the same was observed for genes upregulated upon *mbk-1* loss (*mbk-1* repressed genes) (Fig. 2c, d; Additional file 1: Table S3, 4; Additional file 2: Table S9). In addition, irrespective of genetic background, *mbk-1* induced DEGs significantly overlapped with most to all lists of *daf-16* induced genes in long-lived mutants from six published studies [28–33] (Additional file 1: Table S5). On the other hand, significant overlap with most lists of *daf-16* repressed genes was observed only for *mbk-1* repressed genes from *GSC(−)* worms (Additional file 1: Table S5). Moreover, irrespective of genetic background, *mbk-1* induced genes were commonly enriched for genes associated with the biological processes/gene ontology (GO) terms “organic acid metabolic process” and “defense response to gram-positive bacterium”, although this latter GOterm comprised only very few genes in long-lived mutant backgrounds (Fig. 2e; Additional file 1: Table S6). Conversely, enrichment analysis among *mbk-1* repressed genes did not reveal any GO terms shared across all four backgrounds (Fig. 2f). Interestingly, in *GSC(+)/daf-2(+)* worms, both *mbk-1* induced- and *mbk-1* repressed genes, were enriched for functions in “response to biotic stimulus” and “immune system process” (Fig. 2e, f; Additional file 1: Table S6). In summary, our gene expression- and GO term enrichment analyses pointed towards a partially genetic background-dependent role for *mbk-1* in immunity, reminiscent of a previous study [12], and towards a novel function of *mbk-1* in the regulation of organic acid metabolism.

### *Mbk-1* modulates the expression of fatty acid desaturase genes

**Fig. 3:**
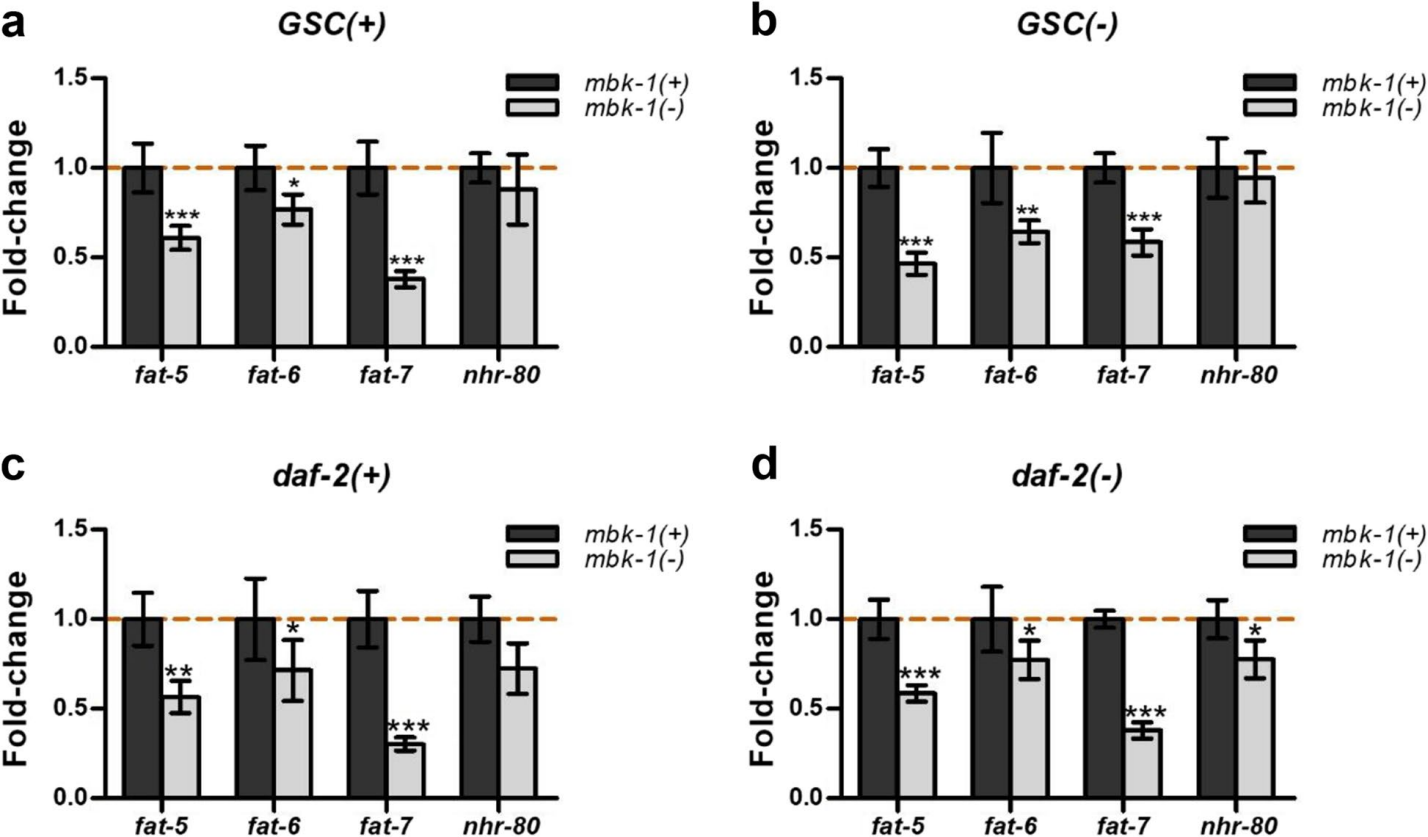
*mbk-1* promotes the expression of fatty acid desaturase-genes*.* Gene expression levels were determined for the genes indicated in *GSC(+)* **(a)**, *GSC(−)* **(b)**, *daf-2(+)* **(c)** and *daf-2(−)* **(d)** by qPCR in day 2 adult worms. Data shown represent mean fold-changes ± SEM in *mbk-1(−)* relative to *mbk-1(+)* worms from four biological replicates. Statistical significance of expression differences was determined by two-way ANOVA with Bonferroni post tests (* *p* < 0.05, ** *p* < 0.01, *** *p* < 0.001)

The GOterm “organic acid metabolic process” showed significant enrichment among *mbk-1* induced genes irrespective of genetic background, and contained two of the three *C. elegans* Δ9-fatty acid desaturase genes [34], *fat-5* and *fat-6*, in *GSC(−)* and in *daf-2(+)* worms (Fig. 2e, Additional file 1: Table S3). Differential expression of the third desaturase, *fat-7*, was not statistically significant in any of the four backgrounds examined (Additional file 2: Table S9). Previous reports indicate that *fat-5; fat-6; fat-7* triple mutants are not viable, and that both, *fat-6* and *fat-7* together, are required for *GSC(−)* longevity, but not for normal wildtype lifespan [35, 36]. Similarly, the *fat-5*/*fat-6/fat-7* transcriptional regulator *nhr-80* is required for *GSC(−)*, but not *daf-2(−)* longevity [35]. Given this preferential requirement of *mbk-1* and Δ9-fatty acid desaturation for *GSC(−)* longevity, we examined the effect of *mbk-1* loss on *fat-5/fat-6/fat-7*- and *nhr-80* expression in *GSC(−)*, *daf-2(−)* and corresponding *GSC(+)/daf-2(+)* control strains by qPCR. These experiments revealed repression of all three Δ9-desaturase genes in *mbk-1(−)* worms of all four backgrounds, while *nhr-80* was suppressed to a statistically significant extent only in *daf-2(−)* worms (Fig. 3). Of note, in agreement with published studies [25–27, 35], *GSC(−)* and *daf-2(−)* worms displayed increased mRNA-levels of *fat-5* and *fat-6*, but not *fat-7* relative to corresponding *GSC(+)* or *daf-2(+)* animals (Additional file 2: Table S9, Additional file 3: Fig. S2). In summary, our qPCR analyses indicated that *mbk-1* positively regulates Δ9-desaturase gene expression and that this regulatory role is independent of *GSC* and *daf-2* status.

### *Mbk-1* modulates the expression of genes implicated in pathogen defense and immunity

**Fig. 4:**
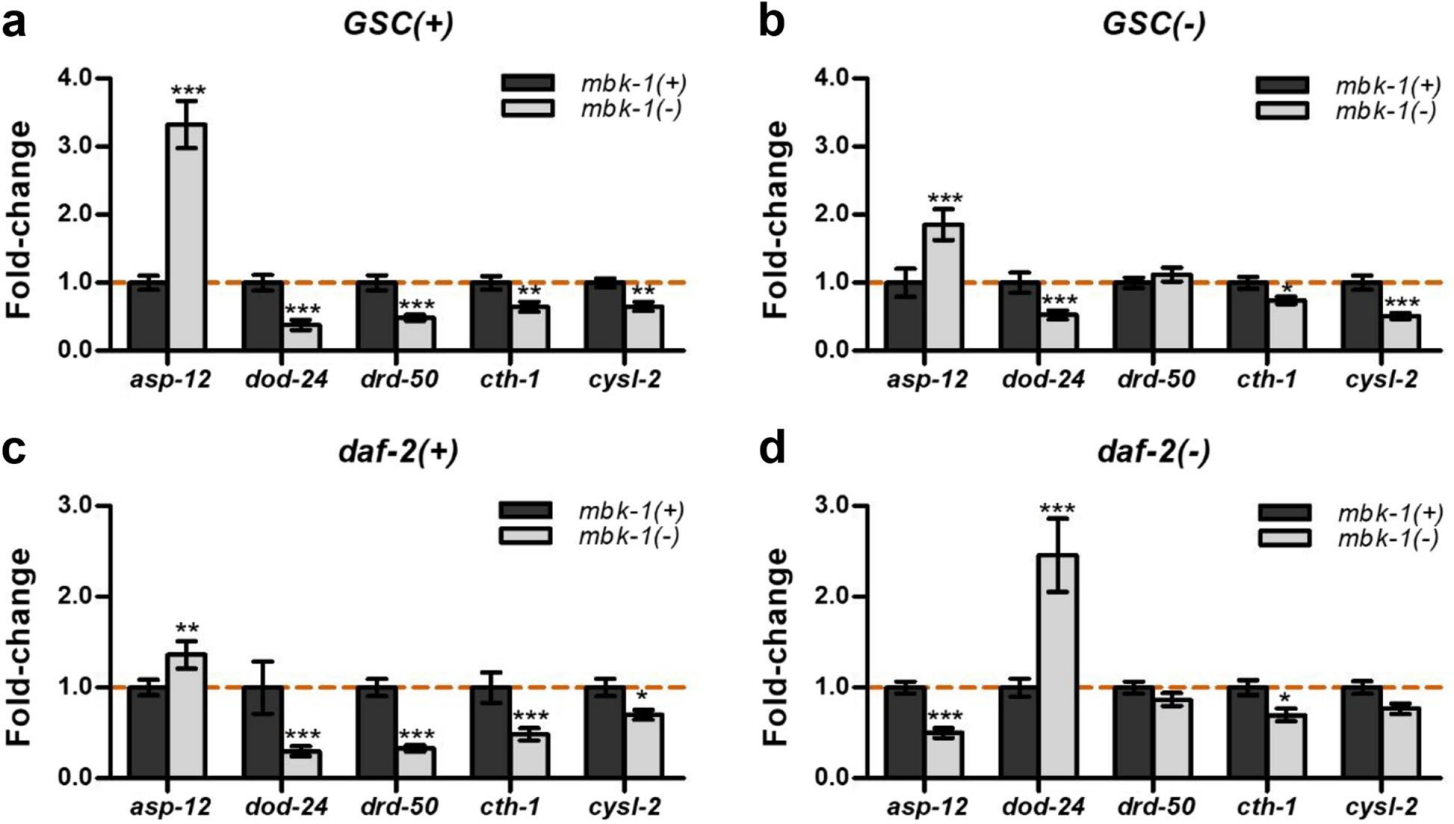
*mbk-1* promotes the expression of pathogen defense- and H_2_S/HCN metabolism genes. Gene expression levels were determined for the genes indicated in *GSC(+)* **(a)**, *GSC(−)* **(b)**, *daf-2(+)* **(c)** and *daf-2(−)* **(d)** by qPCR in day 2 adult worms. Data shown represent mean fold-changes ± SEM in *mbk-1(−)* relative to *mbk-1(+)* worms from four biological replicates. Statistical significance of expression differences was determined by two-way ANOVA with Bonferroni post tests (* *p* < 0.05, ** *p* < 0.01, *** *p* < 0.001)

Our RNA-seq based gene expression- and GO term enrichment-analyses further suggested that *mbk-1* loss disturbs the expression of genes related to pathogen defense/immunity, particularly in *GSC(+)/daf-2(+)* worms (Fig. 2e, f). To further validate this observation, we examined mRNA-levels of selected genes associated with both, “response to biotic stimulus” and the more specialized GOterm “immune system process” by qPCR. In agreement with our RNA-seq data, these qPCR-analyses indicated induction of *asp-12* (a cathepsin E-ortholog) [37], as well as repression of *dod-24* (an epoxide hydrolase 1 ortholog) [32] and *drd-50* (nematode-specific) [37], upon *mbk-1* loss in *GSC(+)/daf-2(+)* worms (Fig. 4a, c; Additional file 1: Table S3). Similarly, qPCR confirmed that *mbk-1* loss causes *asp-12* induction and *dod-24* repression in *GSC(−)*, but not in *daf-2(−)* worms (Fig. 4b, d). Moreover, *drd-50* expression was consistently unaffected by *mbk-1* loss in *GSC(−)* worms (Fig. 4b; Additional file 1: Table S3). Finally, our analyses confirmed previous studies [25–27] that had reported differential expression of *asp-12*, *dod-24* and *drd-50* between *daf-2(−)* and *daf-2(+),* and of *asp-12* between *GSC(−)* and *GSC(+)* worms (Additional file 3: Fig. S3).

As none of the *mbk-1* dependent DEGs validated above has been implicated in the same form of pathogen resistance than *mbk-1* itself has been implicated in, we searched or RNA-seq data for genes that may mediate this known role of *mbk-1*. Specifically, *mbk-1* protects *C. elegans* from fast-killing by a particular strain of the gram-negative bacterium *P. aeruginosa*, PAO1 [12]. Given that growing *C. elegans* in the presence of hydrogen sulfide (H_2_S) also confers PAO1-resistance [38], and that PAO1 kills *C. elegans* by hydrogen cyanide (HCN) poisoning [39], we focused on genes implicated in H_2_S- and/or HCN-metabolism. Indeed, qPCR-analysis indicated *mbk-1* loss-induced downregulation of the cystathionine gamma lyase ortholog *cth-1* [40] in all four backgrounds examined, confirming results or trends also observed in our RNA-seq data (Fig. 4; Additional file 1: Table S3). In addition, a known PAO1-resistance gene, the cyanoalanine synthase *cysl-2* [38] was downregulated upon *mbk-1* loss in *GSC(−)* and in *GSC(+)/daf-2(+) C. elegans* (Fig. 4). Collectively, these data indicate that *mbk-1* modulates the expression of pathogen defense genes in a genetic background-dependent manner, and rise the possibility that *mbk-1*’s function in pathogen resistance/immunity is not limited to fast-killing by gram-negative *P. aeruginosa* PAO1.

## Discussion

In this study, we show that the *DYRK1*-ortholog *mbk-1*, which is required for full longevity of germline-deficient *C. elegans*, also promotes stress resistance in these animals. Moreover, using a transcriptomics approach, we discovered that *mbk-1* modulates the expression of genes implicated in pathogen defense and in fatty acid desaturation, a process that, as *mbk-1*, strongly contributes to *GSC(−)*, but not to *daf-2(−)* longevity. Thus, our study helps explain *mbk-1*’s previously described activities in *C. elegans* lifespan regulation and pathogen resistance. To our knowledge, this report also is the first in any organism to profile the influence of a *DYRK1A*-family member on gene expression in the postmitotic adult stage.

### Genes and pathways deregulated upon loss of *mbk-1*

Our RNA-seq profiling of normal and long-lived *GSC(−)* and *daf-2(−) C. elegans* revealed global changes in gene expression that were more limited in *daf-2(−)* than in *GSC(−)* or *GSC(+)/daf-2(+)* animals, but over-all similar across all backgrounds examined. Thus, it is not surprising that *mbk-1* regulated genes were functionally enriched for some of the same biological processes. Remarkably, *mbk-1* most consistently, i.e. across multiple or all backgrounds analyzed here, modulated the expression of genes implicated in organic acid metabolism and pathogen defense. Indeed, similar GO terms, such as “metabolic process” and “immune system process” were also detected in published gene expression studies on the *mbk-1* ortholog in other systems, including zebrafish embryos [41], and HeLa cells [42]. Collectively, these data provide evidence for *mbk-1/DYRK1A* playing an evolutionarily conserved role in these processes.

### Pathways that may account for *mbk-1*’s particular requirement in germline longevity

Loss of *mbk-1* can decrease Δ9-fatty acid desaturase gene expression in *GSC(−)*, *daf-2(−)* and in *GSC(+)/daf-2(+)* worms. All three *C. elegans* Δ9-fatty acid desaturases, *fat-5*, *fat-6*, and *fat-7*, are orthologous to the human Δ9-desaturases *hSCD1* and *hSCD5* and convert palmitoyl-CoA (*fat-5*) or stearoyl-CoA (*fat-6/7*) into palmitoyl- and oleoyl-CoA, respectively [43, 44]. While wildtype worms tolerate simultaneous inactivation of up to two desaturases with little to no lifespan decrease [35, 36], inactivation of *fat-6*, alone or in combination with *fat-5*, or concomitant inactivation of *fat-6* and *fat-7* results in a moderate to substantial reduction of *GSC(−)* longevity [35]. Therefore, full *GSC(−)* lifespan extension appears to be particularly dependent on *fat-6*, and reduced *fat-6* expression in *GSC(−); mbk-1(−)* double-mutant worms may explain their reduced longevity. In *daf-2(−)* worms, the roles of *fat-5*, *fat-6* and *fat-7* for lifespan extension have not been explicitly examined, even though at least *fat-5* and *fat-6* are positively regulated by *daf-16,* a key factor for *daf-2* longevity [30, 32, 33]. Yet, *nhr-80*, another transcriptional regulator of *fat-5/fat-6/fat-7* [35, 36], is dispensable for *daf-2(−)* lifespan regulation [35]. Thus, it is unclear whether reduced *fat-5/fat-6/fat-7* could also contribute to the slightly reduced longevity of *daf-2(−); mbk-1(−)* worms [13].

### Role of *mbk-1* in pathogen defense

*Mbk-1* has been implicated in the resistance against a particular strain of the gram-negative bacterium *P. aeruginosa*, PAO1 [12], which produces HCN to rapidly kill *C. elegans* [39, 45]. More specifically, *mbk-1* contributes to transcriptional activation of the key PAO1-resistance factor HIF-1, but the precise mechanisms remained unclear [12]. The results we present here suggest a potential solution for the so far “missing link” between *mbk-1* and *hif-1*. Specifically, *mbk-1* promotes the expression of *cth-1*, a cystathionine gamma lyase involved in the synthesis of H_2_S [40]. Moreover, *mbk-1* induces the cyanoalanine synthase *cysl-2*, which directly detoxifies the PAO1 virulence factor HCN [38] in a reaction that also generates H_2_S [38]. H_2_S in turn was proposed as an endogenous activator of HIF-1 [46]. Beyond PAO1-resistance, it is interesting to note that *GSC(−)* worms display elevated levels of H_2_S relative to wildtype *C. elegans* and require transsulfuration pathway enzymes, and by extension: H_2_S, for their longevity [47]. Therefore, it is tempting to speculate that ensuring beneficial levels of H_2_S constitutes another mechanism via which *mbk-1* ensures *GSC(−)* longevity.

Remarkably, we observed that *mbk-1* in wildype *C. elegans* also promotes the expression of genes such as *dod-24* and *drd-50,* which are upregulated upon exposure to another pathogenic *P. aeruginosa* strain, PA14 [37]. PA14, in contrast to PAO1, kills *C. elegans* slowly by gut colonization [12, 37]. Even though some PA14-induced genes such as *asp-12* [37] are suppressed by *mbk-1*, our results provide evidence for a broader function of *mbk-1* in pathogen defense that is not restricted to the previously reported protection from *P. aeruginosa* PAO1 [12]. Clearly, it will be interesting to examine how *mbk-1* loss modulates *C. elegans’* response to other pathogens in the future.

## Conclusions

In summary, the data reported here suggest specific mechanisms underlying previously known activities of the *C. elegans* DYRK1-family kinase *mbk-1* in the regulation of lifespan and pathogen defense. Moreover, they provide further evidence for genetic background-restricted, as well as for genetic background-independent functions of *mbk-1*. Specific novel regulatory influences of *mbk-1* emerging from our studies include fatty acid desaturation, H_2_S-metabolism and *P. aeruginosa* PA14-resistance. As many components of these pathways, and *mbk-1* itself, are evolutionarily conserved, our results may also be applicable to *mbk-1* orthologs in postmitotic tissues of other species.

## Methods

### *C. elegans* strains and culture

Strains used in this study are listed in Additional file 1: Table S7. Worms were cultured following standard protocols on NG agar plates seeded with *E. coli* OP50 [48]. *C. elegans* carrying the *glp-1(e2144ts)*-mutation served as a genetic model for germline-deficiency [15, 47]. To eliminate germ cells, *glp-1(ts)* strains (referred to as *GSC(−)* in Results/Discussion), and corresponding *glp-1(+)* (i.e. *GSC(+)*) control strains, were incubated at 25 °C for the first 24 h of postembryonic development and subsequently, shifted to 20 °C for the remainder of the experiment. *Daf-2(e1370)* worms and corresponding *daf-2(+)* control worms were continuously cultured at 20 °C.

### Stress resistance assays

Worms were synchronized by hypochlorite treatment and transferred to assay plates on day 2 of adulthood at a density of 20–30 worms per 3 cm plate for heat stress experiments and 50–60 worms per 3 cm plate for oxidative stress experiments. Survival was scored every 1–2 h. Heat stress was imposed by incubation at 35 °C. For oxidative stress experiments, assay plates contained 15.4 mM *tert*-butyl hydroperoxide (TBHP) and were prepared 12 h before starting the experiment.

### Growing worms for RNA-extraction

To obtain synchronized populations, gravid adults were treated with hypochlorite and eggs were allowed to hatch in M9 overnight. ~ 700 L1 larvae per strain were plated on 10 cm NG agar plates seeded with concentrated *E. coli* OP50 and cultured at the required temperatures (cf. above). At the L4-stage, 500 worms per strain were transferred to two *E. coli* OP50-seeded 6 cm NG agar plates supplemented with 20 μM FUDR to inhibit germ cell proliferation and progeny production. At day 2 of adulthood, worms were harvested by washing them off their plates with M9. After additional washing with M9 and RNAse-free water, worms were suspended in 1 ml Trizol, snap-frozen in liquid nitrogen and stored at − 80 °C until RNA-extraction.

### RNA-extraction

RNA was extracted using Trizol and cleaned up with the Monarch® Total RNA Miniprep Kit or RNA Cleanup Kit (New England Biolabs) according to the manufacturer’s instructions.

### RNA-sequencing

RNA quality control, library preparation, and 50 bp single-end RNA-sequencing on the Illumina HiSeq4000 platform was performed at Eurofins Genomics, Ebersberg, Germany. All samples were analyzed in 3 biological replicates and a minimum of 33.2 * 10^6^ reads (maximum 49.9 * 10^6^, median 39.7 * 10^6^ reads) per replicate were obtained, resulting in at least 16.5x genome coverage (maximum 24.9x, median 20x). A minimum of 91.4% of reads (maximum 96.9%, median 95.7%) could be mapped to the *C. elegans* reference genome (cf. below).

### RNA-seq data analysis

Tools provided at https://usegalaxy.eu/ were used for initial analysis steps: *FastQC* (v0.72) and *MultiQC* (v1.7) for initial quality control; *Trimmomatic* (v0.36.5; ILLUMINACLIP with default settings, LEADING:20, TRAILING:20, SLIDINGWINDOW:5:20, MINLENGTH:20) for gentle trimming of reads; *STAR* (v2.7.2a) for alignment to the *C. elegans* reference genome (Wormbase release WS271); and *featureCounts* (v1.6.4) for read summarization [49–54]. One replicate of the *daf-2(+); mbk-1(−)* strain was excluded at this stage. Subsequent analyses were performed using Bioconductor (v3.8–3.12) packages and custom R scripts (v 3.5.3–4.0.4) [55]. Differential expression analysis with *DESeq2* (v1.22.2–1.30.1) [56] was limited to genes detected with at least one count in each biological replicate of at least one of the strains analyzed. Genes with less than 10 counts across the samples compared were removed by pre-filtering. RNA-seq data was visualized with MA- and Volcano-plots using the *apeglm*-method in *DESeq2* for shrinking log-fold-changes [57], and the R/Bioconductor-package *EnhancedVolcano* (v1.0.1–1.8.0). Differentially expressed genes (DEGs) were defined as p_adj_ (Benjamini-Hochberg adjusted *p*-value/FDR) < 0.05 in *DESeq2*-analysis and expression fold-change > 1.5/ < 0.6667 (|Log_2_FC| > 0.58).

### Overlap of DEG-lists

Overlaps between lists of DEGs were visualized using the *Venn and Euler Diagrams*-App in *Cytoscape* (v3.8.2) [58]. Statistical significance of overlaps was calculated as the hypergeometric probability of detecting at least as many common genes as observed in the two lists, using the *phyper*-function in R. Representation factors were calculated as the number of overlaping genes divided by the expected number of overlapping genes in the two lists (http://nemates.org/MA/progs/overlap_stats.html; last accessed 12. April 2019). For all calculations, the number of genes in the genome was set to 18,980, i.e. the number of genes in Wormbase WS271 that passed low-count filtering during *DESeq2*-analysis (cf. above). DEG-lists from published studies were, if necessary, converted to current WBGene-IDs using WormMine (http://intermine.wormbase.org/tools/wormmine/begin.do; last accessed 04. September 2019) and adjusted to genes in Wormbase WS271 using custom R-scripts and manual curation.

### GO term enrichment analysis of DEG lists

GO term enrichment analysis was performed using the enrichment tool provided by Wormbase (https://wormbase.org/tools/enrichment/tea/tea.cgi; last accessed: 31 March 2021) [59]. The q-value threshold was set to < 0.05 unless otherwise stated. GO term categories were retrieved using REViGO ([60] http://revigo.irb.hr/; last accessed 31 March 2021).

### qPCR

One microgram total RNA was reverse-transcribed using LunaScript® RT SuperMix (New England Biolabs). qPCR-reactions were performed in 2–3 technical replicates in 20 μl reaction volume on an CFX Connect™ Real-Time PCR Detection System (Bio-Rad Laboratories) with iTaq™ Universal SYBR® Green Supermix (Bio-Rad Laboratories). The thermal cycling protocol comprised one activation step at 95 °C for 3 min, followed by 40 cycles of denaturation at 95 °C for 10 s and combined annealing/extension at 60 °C for 30 s. Melting curve analysis was performed from 65 °C to 95 °C with 0.5 °C increments at 5 s per step. Data were analyzed by the ΔΔCt method and target gene expression levels were normalized to the geometric mean of *cdc-42*, *tba-1* and *Y45F10D.4* [61, 62]. Primer sequences are listed in Additional file 1: Table S8.

### Statistical analysis

Statistical analysis was performed using Prism 5 (GraphPad Software, San Diego, CA, USA). Details on the particular tests used are specified in the figure legends.

## Supplementary Information

**Additional file 1: Table S1.** Statistical analysis of stress resistance data. Accompanies Fig. 1. Worms of the strains indicated were subjected to heat or oxidative stress as specified in the individual table headlines. For each biological replicate of the respective experiment, the table lists mean and median survival times in h, standard deviations and standard errors of the mean, the number of worms scored, and *p*-values from Kaplan-Meier survival analysis with Mantel-Cox tests against the control-, *daf-16(−)* or *mbk-1(−)* strain of the same genetic background. In case of the *GSC(−)* and *daf-2(−)* control strain, survival analysis was performed relative to the *GSC(+)* or *daf-2(+)* control strain. Repeated measures two-way ANOVA with Bonferroni post tests was performed on the mean survival times determined in the individual biological replicates of the experiments. Statistically significant *p*-values (* *p* < 0.05, ** *p* < 0.01, *** *p* < 0.001) are highlighted in blue. **Table S2.** Genes regulated by *daf-2(−)* and *GSC(−)* in this work and in published studies. For each overlap, the number of genes shared between the two particular lists, the representation factor, and the p-value (in italics; hypergeometric probability) are indicated. Statistically significant overlaps (*p* < 0.05) are highlighted in blue. For these overlap analyses, lists of published studies were adjusted to WS271 and limited to genes that passed low-count filtering in our *DESeq2*-analyses. (cf. Methods). See main text for full references of the published studies included in the table. **Table S3.** Genes differentially expressed between *mbk-1(−)* and *mbk-1(+)* worms of the genetic backgrounds analyzed in this study. Differentially expressed genes (DEGs) were defined by p_adj_ < 0.05 in *DESeq2*-analysis and by the following expression fold-change thresholds: FC < 0.6667 (Log_2_FC < − 0.58) for genes downregulated upon *mbk-1* loss. Expression of these genes is normally promoted by *mbk-1* (*mbk-1* induced DEGs). FC > 1.5 (Log_2_FC > 0.58) for genes upregulated upon *mbk-1* loss. Expression of these genes is normally repressed by *mbk-1* (*mbk-1* repressed DEGs). In addition, the table lists DEGs detected in control comparisons between the following strains (all *mbk-1(+)*): downregulated < 0.6667-fold or upregulated > 1.5-fold in *daf-2(−)* relative to *daf-2(+)*; downregulated < 0.25-fold (Log_2_FC < − 2) or upregulated > 4-fold (Log_2_FC > 2) in *GSC(−)* relative to *GSC(+).* See Additional file 2 for full *DESeq2*-data. **Table S4.** Overlaps of *mbk-1* dependent DEGs in the genetic backgrounds analyzed in this study. Accompanies Fig. 2c, d. (a) Pairwise overlaps between DEG-lists. For each overlap, the number of genes shared between the two particular lists, the representation factor, and the p-value (in italics; hypergeometric probability) are indicated. Statistically significant overlaps are highlighted in blue. (b) Absolute numbers of *mbk-1* dependent DEGs that are induced/repressed exclusively in the background indicated, or that are also induced/repressed in one or more of the three other backgrounds. (c) Percentages of *mbk-1* dependent DEGs that are induced/repressed exclusively in the background indicated, or that are also induced/repressed in one or more of the three other backgrounds. **Table S5.** Overlap of genes regulated by *mbk-1* in this work and by *daf-16* in published studies. For each overlap, the number of genes shared between the two particular lists, the representation factor, and the p-value (in italics; hypergeometric probability) are indicated. Statistically significant overlaps (*p* < 0.05) are highlighted in blue. For these overlap analyses, lists of published studies were adjusted to WS271 and limited to genes that passed low-count filtering in our *DESeq2*-analyses. (cf. Methods). See main text for full references of the published studies included in the table. **Table S6.** GO term-enrichment among *mbk-1* dependent DEGs in the genetic backgrounds analyzed in this study. Accompanies Fig. 2e, f. *mbk-1* induced and -repressed DEGs from the backgrounds indicated were subjected to GO term enrichment analysis. q-values < 0.05 were considered statistically significant. The table covers all three GO term categories (biological process [bp], cellular component [cc] and molecular function [mf]), while only terms of the bp-category were plotted in Fig. 2e, f. Note that Fig 2e, f are further limited to GO terms of the bp-category with N_observed_ ≥ 2 and enrichment fold-change ≥ 2 and that some GO terms were not plotted in these figures for the reasons specified in the Comment-column. **Table S7.** List of *C. elegans* strains used in this study. **Table S8.** List of qPCR-primers used in this study.

**Additional file 2: Table S9.** *DESeq2*-analysis of the RNA-seq data set generated in this study. Differential gene expression was determined between *mbk-1(−)* and *mbk-1(+)* worms (abbreviated as “-VS+” in column headers) in *GSC(+)*, *GSC(−)*, *daf-2(+)* and *daf-2(−)* background. In addition, control comparisons were made between the *mbk-1(+)* strains of the *daf-2(−)* and *daf-2(+)*, and the *GSC(−)* and *GSC(+)* backgrounds. The table shows *DESeq2*-results for all genes that passed our filter criteria in at least one of the comparisons (cf. Methods).

**Additional file 3: Fig. S1.** Global changes in gene expression upon *daf-2* or GSC loss*.* Accompanies Fig. 2. (a and b) Differential gene expression between the strains indicated was determined by RNA-seq and visualized in MA-plots (a) and Volcano plots (b). Light blue lines indicate |Log_2_FC| > 0.58 and p_adj_ < 0.05, open triangles indicate data points beyond axis limits. See Additional file 2: Table S9 for complete *DESeq2*-analysis results. **Fig. S2.** Influence of *GSC* and *daf-2* status on fatty acid desaturase gene-expression*.* Accompanies Fig. 3. Expression levels of the genes indicated were determined in *GSC(−)* relative to *GSC(+)* (a), and in *daf-2(−)* relative to *daf-2(+)* worms (b) by qPCR in day 2 adults. Data shown represent mean fold-changes ± SEM from four biological replicates. Statistical significance of expression differences was determined by two-way ANOVA with Bonferroni post tests (* *p* < 0.05, ** *p* < 0.01, *** *p* < 0.001). Note that other authors [25] have proposed a fold-change of ~4 for detecting genes differentially expressed between *GSC(+)* and *GSC(-)* worms, as they differ in the presence of a germline. The following gene expression changes (or lack of change) relative to the corresponding *GSC(+)/daf-2(+)* strain were also observed in published studies: *GSC(−)*: induction of *fat-5/nhr-80* [25, 35], induction of *fat-6* [35], no upregulation of *fat-7* (our qPCR, [25]) or downregulation of fat-7 (our RNA-seq, [35]); *daf-2(−)*: induction of *fat-5* [26, 27] and *fat-6* [26], repression of *fat-7* [26, 27], no differential expression of *nhr-80* [26, 27]. **Fig. S3.** Influence of *GSC* and *daf-2* status on the expression of genes involved in pathogen defense and H_2_S/HCN metabolism*.* Accompanies Fig. 4. Expression levels of the genes indicated were determined in *GSC(−)* relative to *GSC(+)* (a), and in *daf-2(−)* relative to *daf-2(+)* worms (b) by qPCR in day 2 adults. Data shown represent mean fold-changes ± SEM from 3 to 4 biological replicates. Statistical significance of expression differences was determined by two-way ANOVA with Bonferroni post tests (* *p* < 0.05, ** *p* < 0.01, *** *p* < 0.001). Note that other authors [25] have proposed a fold-change of ~ 4 for detecting genes differentially expressed between *GSC(+)* and *GSC(−)* worms, as they differ in the presence of a germline. The following gene expression changes (or lack of change) relative to the corresponding *GSC(+)/daf-2(+)* strain were also observed in published studies: *GSC(−)*: significant induction of *asp-12* [25] (*GSC(−)* repressed genes not published in this study, thus it is unclear whether *dod-24*, *drd-50, cth-1* and *cysl-2* were not induced/induced < 4-fold, or even repressed by GSC loss); *daf-2(−)*: significant induction of *asp-12*, significant repression of *dod-24* and *drd-50*, no differential expression of *cysl-2* [26, 27]; no differential expression of *cth-1* in [27], but repression in [26].

## Data Availability

The RNA sequencing data set generated during the present study has been deposited in the ArrayExpress database at EMBL-EBI (www.ebi.ac.uk/arrayexpress) under accession number E-MTAB-10898. Complete results from the DESeq2-analysis are also included in this article in Additional file 2: Table S9.

## Abbreviations

DEGs: Differentially expressed genes
FC: Fold-change
GO term: Gene ontology term
GSCs: Germline stem cells
RNA-seq: RNA-sequencing
SEM: Standard error of the mean
TBHP: Tert-butyl hydroperoxide
