# Supplementary material for: Regulation of fatty acid desaturase- and immunity gene-expression by mbk-1/DYRK1A in Caenorhabditis elegans
Source: BMC Genomics. 2022 Jan 4;23:25. doi: 10.1186/s12864-021-08176-y (PMC8729107; doi:10.1186/s12864-021-08176-y)
Supplement: Supplementary file 3 — Additional file 3: Fig. S1. Global changes in gene expression upon daf-2 or GSC loss. Accompanies Fig. 2. (a and b) Differential gene expression between the strains indicated was determined by RNA-seq and visualized in MA-plots (a) and Volcano plots (b). Light blue lines indicate |Log2FC| > 0.58 and padj < 0.05, open triangles indicate data points beyond axis limits. See Additional file 2: Table S9 for complete DESeq2-analysis results. Fig. S2. Influence of GSC and daf-2 status on fatty acid desaturase gene-expression. Accompanies Fig. 3. Expression levels of the genes indicated were determined in GSC(−) relative to GSC(+) (a), and in daf-2(−) relative to daf-2(+) worms (b) by qPCR in day 2 adults. Data shown represent mean fold-changes ± SEM from four biological replicates. Statistical significance of expression differences was determined by two-way ANOVA with Bonferroni post tests (* p < 0.05, ** p < 0.01, *** p < 0.001). Note that other authors [25] have proposed a fold-change of ~4 for detecting genes differentially expressed between GSC(+) and GSC(-) worms, as they differ in the presence of a germline. The following gene expression changes (or lack of change) relative to the corresponding GSC(+)/daf-2(+) strain were also observed in published studies: GSC(−): induction of fat-5/nhr-80 [25, 35], induction of fat-6 [35], no upregulation of fat-7 (our qPCR, [25]) or downregulation of fat-7 (our RNA-seq, [35]); daf-2(−): induction of fat-5 [26, 27] and fat-6 [26], repression of fat-7 [26, 27], no differential expression of nhr-80 [26, 27]. Fig. S3. Influence of GSC and daf-2 status on the expression of genes involved in pathogen defense and H2S/HCN metabolism. Accompanies Fig. 4. Expression levels of the genes indicated were determined in GSC(−) relative to GSC(+) (a), and in daf-2(−) relative to daf-2(+) worms (b) by qPCR in day 2 adults. Data shown represent mean fold-changes ± SEM from 3 to 4 biological replicates. Statistical significance of expression differen [file 12864_2021_8176_MOESM3_ESM.pdf]

Additional file 3

See main text for references.

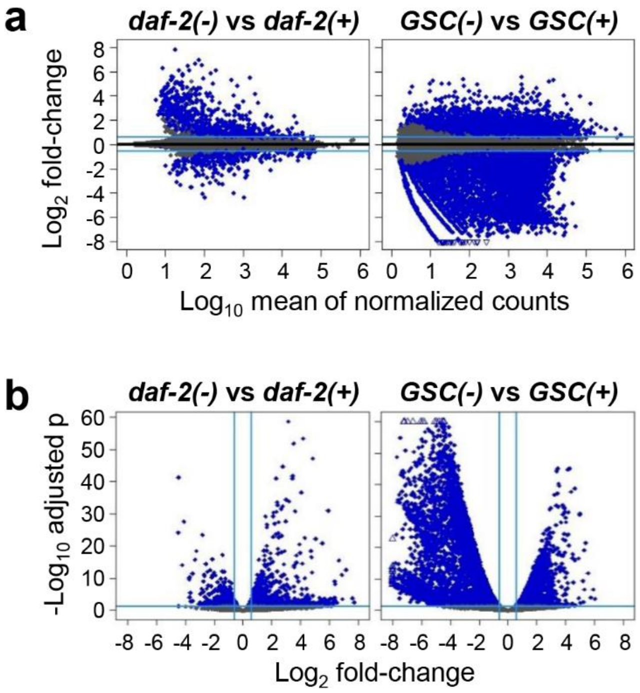

Fig. S1 Global changes in gene expression upon *daf-2* or GSC loss*.* Accompanies Fig. 2. **(a** and **b)** Differential gene expression between the strains indicated was determined by RNA-seq and visualized in MA-plots **(a)** and Volcano plots **(b)**. Light blue lines indicate $|Log_2FC| > 0.58$ and $p_{adj} < 0.05$, open triangles indicate data points beyond axis limits. See Additional file 2: Table S9 for complete *DESeq2*-analysis results.

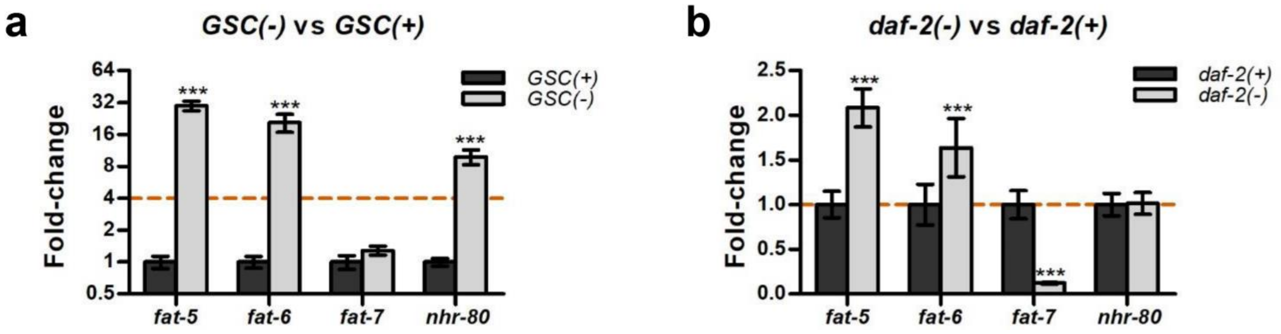

Fig. S2 Influence of *GSC* and *daf-2* status on fatty acid desaturase gene-expression*.* Accompanies Fig. 3. Expression levels of the genes indicated were determined in *GSC(-)* relative to *GSC(+)* **(a)**, and in *daf-2(-)* relative to *daf-2(+)* worms **(b)** by qPCR in day 2 adults. Data shown represent mean fold-changes ± SEM from four biological replicates. Statistical significance of expression differences was determined by two-way ANOVA with Bonferroni post tests (* $p < 0.05$, ** $p < 0.01$, *** $p < 0.001$). Note that other authors [25] have proposed a fold-change of ~4 for detecting genes differentially expressed between *GSC(+)* and *GSC(-)* worms, as they differ in the presence of a germline. The following gene expression changes (or lack of change) relative to the corresponding *GSC(+)/daf-2(+)* strain were also observed in published studies: *GSC(-)*: induction of *fat-5/nhr-80* [25, 35], induction of *fat-6* [35], no upregulation of *fat-7* (our qPCR, [25]) or downregulation of *fat-7* (our RNA-seq, [35]); *daf-2(-)*: induction of *fat-5* [26, 27] and *fat-6* [26], repression of *fat-7* [26, 27], no differential expression of *nhr-80* [26, 27].

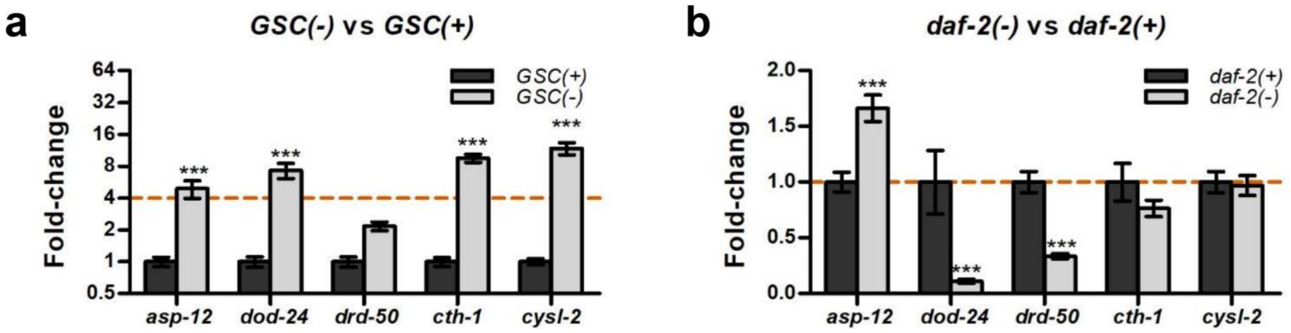

Fig. S3 Influence of *GSC* and *daf-2* status on the expression of genes involved in pathogen defense and H_2S/HCN metabolism. Accompanies Fig. 4. Expression levels of the genes indicated were determined in *GSC(-)* relative to *GSC(+)* **(a)**, and in *daf-2(-)* relative to *daf-2(+)* worms **(b)** by qPCR in day 2 adults. Data shown represent mean fold-changes ± SEM from 3-4 biological replicates. Statistical significance of expression differences was determined by two-way ANOVA with Bonferroni post tests (* p < 0.05, ** p < 0.01, *** p < 0.001). Note that other authors [25] have proposed a fold-change of ~4 for detecting genes differentially expressed between *GSC(+)* and *GSC(-)* worms, as they differ in the presence of a germline. The following gene expression changes (or lack of change) relative to the corresponding *GSC(+)/daf-2(+)* strain were also observed in published studies: *GSC(-)*: significant induction of *asp-12* [25] (*GSC(-)* repressed genes not published in this study, thus it is unclear whether *dod-24*, *drd-50, cth-1* and *cysl-2* were not induced, induced < 4-fold, or even repressed by GSC loss); *daf-2(-)*: significant induction of *asp-12*, significant repression of *dod-24* and *drd-50*, no differential expression of *cysl-2* [26, 27]; no differential expression of *cth-1* in [27], but repression in [26].
